# Anakinra as a diagnostic challenge and treatment option for systemic autoinflammatory disorders of undefined genetic cause

**DOI:** 10.1186/1546-0096-13-S1-P189

**Published:** 2015-09-28

**Authors:** S Harrison, S Nizam, M McDermot, D McGonagle, S Savic

**Affiliations:** 1 University of Leeds, Institute of Rheumatic and Musculoskeletal Medicine, Leeds, UK; 2 Pinderfields Hospital, Department of Rheumatology, Wakefield, UK; 3 Leeds Teaching Hospitals NHS Trust, Clinical Immunology and Allergy, Leeds, UK

## Background

Diverse monogenic autoinflammatory diseases share responsiveness to interleukin (IL)-1 blockade. This study explored the utility of anakinra (an IL-1 receptor antagonist) as a treatment option for clinically heterogeneous systemic inflammatory disease with autoinflammatory presentations where a genetic cause was not defined.

## Methods

A total of ten adult cases with ongoing inflammatory episodes, where alternative diagnoses, including malignancy and infection, were evaluated. Genetic screening was also performed to exclude known genetic causes of autoinflammatory disorders (e.g. cryopyrin-associated periodic syndromes (CAPS), tumour necrosis factor receptor-associated periodic syndrome (TRAPS), etc.)

## Results

All patients had presentations that were atypical of recognised autoinflammatory disorders and all were negative on genetic screening. Eight of ten cases showed rapid responsiveness to anakinra with the ability to subsequently taper alternative immunosuppression. Good responses to anakinra were maintained with inadvertent drug discontinuation being linked to disease flares.

## Conclusions

The spectrum of poorly defined clinical and genetic autoinflammatory disorders that show responsiveness to anakinra is considerable. In fact, responsiveness to anakinra appears to be useful in diagnosis given the characteristically rapid onset of efficacy and symptomatic improvement.

